# A red-NIR fluorescent dye detecting nuclear DNA G-quadruplexes: *in vitro* analysis and cell imaging†
†Electronic supplementary information (ESI) available. See DOI: 10.1039/c6cc08492c



**DOI:** 10.1039/c6cc08492c

**Published:** 2017-01-23

**Authors:** F. Doria, M. Nadai, M. Zuffo, R. Perrone, M. Freccero, S. N. Richter

**Affiliations:** a Dept. of Chemistry , University of Pavia , V.le Taramelli 10 , 27100 Pavia , Italy . Email: mauro.freccero@unipv.it; b Dept. of Molecular Medicine , University of Padua , via Gabelli 63 , 35121 Padua , Italy . Email: sara.richter@unipd.it

## Abstract

Aggregation, red-NIR emission and light-up upon nucleic acid G-quadruplex binding have been investigated for a prototype core-extended naphthalene diimide, which is capable of fast cellular entry and nucleolar localization. Both high-level colocalization with an anti-G-quadruplex antibody and nucleolin displacement reveal that the compound targets and thus makes visible nuclear DNA G-quadruplexes.

## Abstract 2

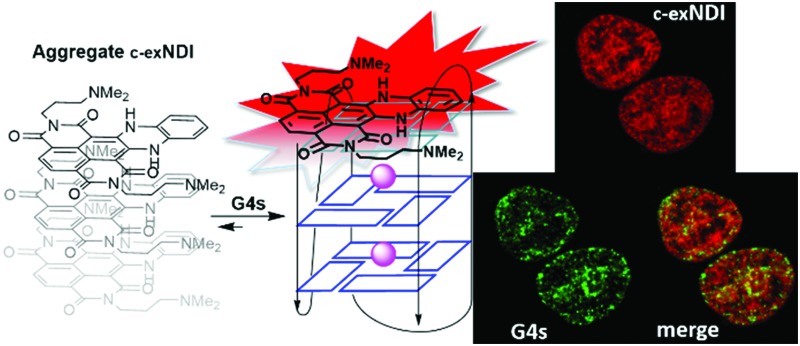
Light-up of nuclear G-quadruplex DNA in cells by an aggregating and red/NIR emitting dye.

G-quadruplexes (G4s) are unique four-stranded nucleic acid structures formed by guanine-rich sequences. Based on mutual strand orientation, they can adopt three main topologies, *i.e.* parallel, antiparallel and hybrid-type. G4s were shown to be involved in key regulatory and pathological roles both in eukaryotes and in microorganisms, including transcriptional regulation of gene promoters and enhancers, translation, chromatin epigenetic regulation, and DNA recombination.^1–5^ Formation of G4s *in vivo* has been substantiated by the discovery of cellular proteins that specifically recognize G4s^6,7^ and development of G4 specific antibodies.^8,9^

Given the biological significance of G4s, extensive efforts by many groups have resulted in a large number of G4-stabilizing ligands as potential inhibitors of pathological processes, such as cancer cell growth,^10,11^ bacterial and viral infections^12–18^ and neurological degeneration.^19^ In line with these potential applications, G4 tracking by small molecule probes, such as fluorescent ligands, has become an equally important research field. In this direction, a number of compounds fluorescing upon G4 binding have been developed.^20–22^ Some of them were able to preferentially recognize definite G4 topologies.^23–25^ A major limitation to their use *in vivo*, however, is their cellular and subcellular permeability. For example, compounds that do not or poorly enter the nucleus are available only for cytoplasmic RNA G4 probing.^26,27^ In addition, compounds must be selective for G4 nucleic acids. For example, Thioflavin T,^28^ which also binds amyloid aggregates, cannot be used for *in vivo* imaging.^29^ Tri- and tetra-substituted naphthalene diimides (NDIs) are potent and reversible ligands,^30,31^ as well as alkylating agents targeting guanine-rich nucleic acids (NAs) folded into G4s.^32,33^ Their performance as cellular fluorescent probes has been implemented by loss of structural planarity,^34^ conjugation to a second NDI unit^35^ or to a coumarin absorbing antenna,^36^ and extension of the aromatic core.^37^ Core-extended NDIs (**c-exNDIs**, Scheme 1) are potent G4 binders, displaying anti-HIV-1 activity due to their ability to bind viral G4s with higher affinity than the cellular G4s.^12^ Nonetheless, because of the high potency of **c-exNDIs**, cellular G4s are also bound with good efficiency.^12^ In addition, the extended aromatic system confers high absorptivity and emission in the red-NIR region to the **c-exNDIs**. These features prompted us to characterise the fluorescence behaviour of the unsubstituted **c-exNDI** (R=H) both in solution and when bound to G4s.

**Scheme 1:**
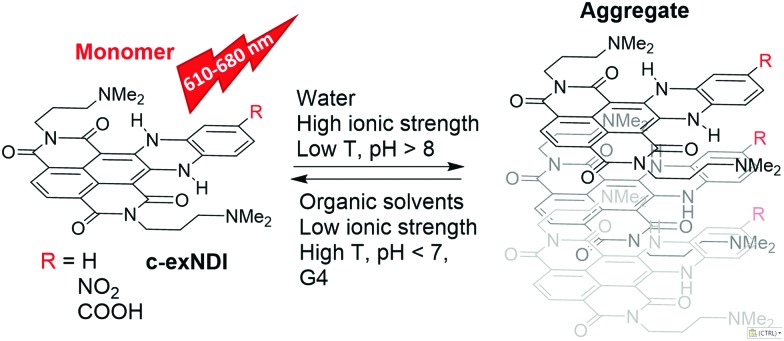
Structures of emitting and aggregating **c-exNDIs**.

The UV-vis spectra of **c-exNDI** in organic solvents (Fig. S1, ESI†) including THF (Fig. 1) showed three absorption bands with the highest peak at 578 nm, indicating the presence of non-aggregated **c-exNDI** monomers. In contrast, the spectrum in water showed two broader peaks at 555 and 605 nm, with a tail up to 700 nm (Fig. 1). **c-exNDI** mirrors the absorbance behaviour of perylene bisimides (**PDIs**), which has been associated with aggregation.^38^ Increasing water in the THF/mixtures, we observed the progressive formation of a **c-exNDI** aggregate (Fig. S2, ESI†). It is known that **PDI** aggregation in water causes significant fluorescence quenching. As expected, the fluorescence intensity of **c-exNDI** (5 × 10^–6^ M) in water was only about 8% of that in THF. Temperature and pH effects on both absorption and emission spectra further corroborated the aggregation evidence (Fig. S3 and S4, ESI†).

**Fig. 1:**
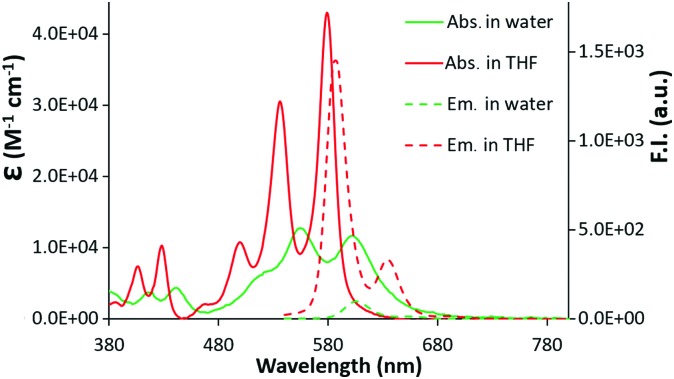
Absorption (2 × 10^–5^ M) and emission (5 × 10^–6^ M, *λ*_exc_ = 500 nm) spectra of a solution of **c-exNDI** in water (green line) and in THF (red line).

To assess whether **c-exNDI** aggregation was also effective in physiological conditions (10 mM Tris–HCl pH 7.4, 100 mM KCl), we measured the compound absorption spectra at increasing concentrations (1.5 × 10^–5^– 3 × 10^–4^ M, Fig. S5, ESI†). Molar absorptivity was found to isodesmically depend on the concentration (*K*_–d_ = 1.1 ± 0.3 × 10^5^ M^–1^). To clarify the difference in the emission properties of the free *vs.* aggregated **c-exNDI**, absorption and excitation spectra were measured in THF and water solution. The spectra were superimposable in THF, while remarkably different in water, with the excitation spectrum exhibiting a profile more similar to that recorded in THF than to that of the absorption spectrum (Fig. S6, ESI†). This suggests that the monomeric form is the only emitting species. We thus decided to investigate whether G4 binding induced disaggregation and consequent light-up. We titrated diluted solutions of **c-exNDI** (5 × 10^–6^ M) with a small NA library (Table S1, ESI†) composed of three anti-parallel G4s (HRAS, hTel22 in Na^+^ and TBA), a hybrid G4 (hTel22 in K^+^), three parallel G4s (c-kit1, c-kit2 and c-myc) and controls (ssDNA and dsDNA). Titrations were performed in both absorption and emission modes. Titration of **c-exNDI** with hTel22 in K^+^ solution induced a red shift in both absorption (15 nm) and emission (12 nm) and signal intensity enhancement (Fig. 2a and b). hTel22 in K^+^ yielded the most intense fluorescence enhancement. With the other NAs, after an initial quenching, we observed a moderate and differential light-up (Fig. 2c). The one exception was dsDNA, with which we measured a progressive quenching of the emission. The fluorescence quantum yields (*Φ*_f_) of **c-exNDI** in the presence of one equivalent of each NA (Table S2, ESI†) increased differently (*i.e.*, from *Φ*_f_ = 24% with hTel22 in K^+^, to *Φ*_f_ = 14% with c-myc) with respect to **c-exNDI** alone (8%), indicating an increase in the monomer content of the solution upon G4 binding. Fitting of fluorescence and absorption data for the different **c-exNDI**/G4 mixtures (Fig. S7, ESI†) allowed us to determine the best complexation models and the apparent binding constants (Table S3, ESI†). These span from 3.43 ± 0.3 × 10^7^ M^–1^ (c-myc) to 1.2 ± 0.5 × 10^5^ M^–1^ (TBA) in the best fitting 1 : 1 model. hTel22 in Na^+^ and HRAS yielded 3.0 ± 0.3 × 10^11^ and 3.0 ± 0.3 × 10^10^ M^–2^ values, respectively, in the 2 : 1 (**c-exNDI** : NA) model. **c-exNDI** : NA stoichiometries were confirmed by Job plot analysis of emission data (for hTel22 see the inset of Fig. 2b and for other NAs see Fig. S8, ESI†). Interestingly, we measured an apparent binding constant of 7.41 ± 0.03 × 10^3^ M^–1^ for dsDNA, in the 1 : 1 model, confirming **c-exNDI** selectivity for the G4 conformation. The ssDNA binding constant (2.85 ± 0.01 × 10^5^ M^–1^, 1 : 1 model), being much lower than those measured for c-myc and hTel22 in K^+^ (120 and 20 fold, respectively), did not limit the probe applicability.

**Fig. 2:**
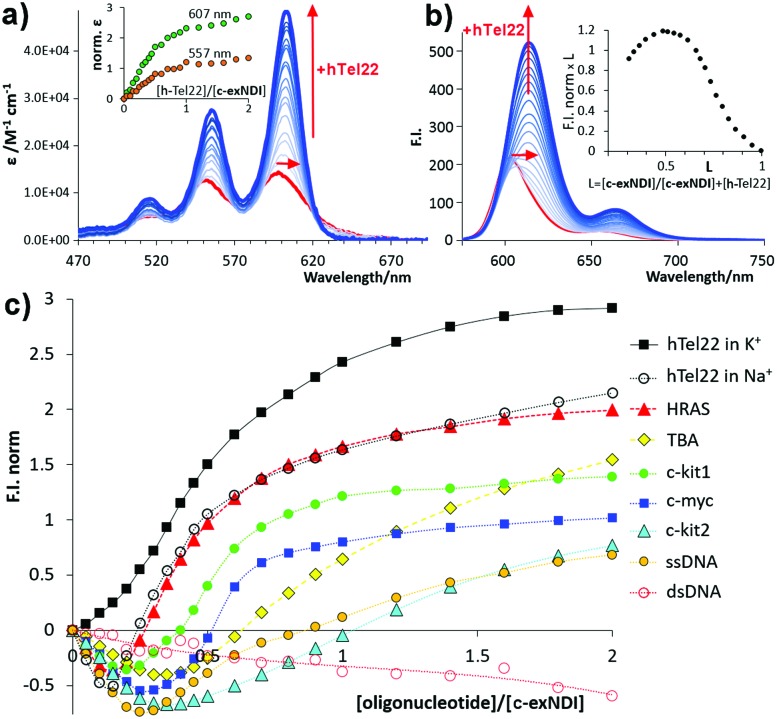
(a) Titration absorption spectra of a 5.0 × 10^–6^ M **c-exNDI** solution (1 × 10^–1^ M KCl, 1 × 10^–2^ M Tris–HCl, pH 7.4) upon addition of hTel22 G4 (2.5 × 10^–7^–1.0 × 10^–5^ M); inset: molar absorptivity enhancement (norm. *ε* = *ε*/*ε*_0_ – 1). (b) Titration emission spectra (*λ*_exc_ = 600 nm) measured under the same conditions as (a). Inset: Job plot analysis of emission data. (c) Fluorescence enhancement factors (F.I._norm_ = *F*/*F*_o_ – 1), plotted as a function of the [oligonucleotide]/[**c-exNDI**] ratio for the titrations of **c-exNDI** with the NAs in Table S1 (ESI†).

Direct interaction of **c-exNDI** with hTel22 G4 was probed by CD and Taq polymerase stop assay analysis. When complexed with the G4, the compound acquired an induced CD (ICD) signal at wavelengths corresponding to its absorption maxima (Fig. S9A, ESI†). When added to the G4-forming template, **c-exNDI** inhibited Taq polymerase progression in a concentration-dependent manner (Fig. S9B and C, ESI†). These data indicate that **c-exNDI** directly interacts with the G4.

These data prompted us to investigate the ability of **c-exNDI** to enter into cells (293T, human embryonic kidney cell line) by UV/vis absorption and fluorescence microscopy. Interestingly, when **c-exNDI**-treated cells were imaged for fluorescence, most of the signal was concentrated in the cell nucleus, with peaks in subnuclear compartments. In contrast, no significant fluorescence was observed in the cytoplasm (Fig. S10A, panels c and d, ESI†). Comparing cells incubated with **c-exNDI** for different time intervals, the fluorescence signal was found to be appreciable in the nucleus only after 2.5 min, demonstrating a very fast cellular and nuclear entry (Fig. S10B, ESI†). This indicates a remarkably different cellular entry and distribution of **c-exNDI** in comparison with recently described fluorogenic probes effectively targeting cellular G4s.^26,39^ To characterize the nuclear localization of **c-exNDI**, treated cells were visualized using confocal microscopy. The compound appeared clustered in discrete foci all over the nucleus, with enhanced assemblage around and inside subnuclear organelles (Fig. S11, panel a, ESI†). Following the *in vitro* observation of **c-exNDI’s** high selectivity for G4 DNA^12^ and effective light-up when bound to human telomeric hTel22 G4, we treated cells with either DNase or RNase to confirm the nature of the main binding target of the compound. RNase treatment did not modify **c-exNDI** nuclear staining/localization (Fig. S11, panel b, ESI†), while the use of DNase profoundly affected the **c-exNDI** signal, largely decreasing it in the nucleoplasm (Fig. S11, panel c, ESI†). Subnuclear localization was maintained, though at lower intensity (Fig. S11, panel c, ESI†), probably due to the inability of DNase to reach the subnuclear organelles. These data indicate that **c-exNDI** in cells mainly binds DNA and that disruption of the **c-exNDI**/DNA complex highly impairs compound fluorescence. To check whether DNA G4s were the preferred targets not only *in vitro* but also in cells, cells were incubated with **c-exNDI**, washed, fixed and treated with the 1H6 antibody,^8^ specifically selected to recognize DNA G4 structures *in vitro* and in cells.^8,40^ Indeed, we observed a good colocalization of **c-exNDI** and 1H6 (Fig. 3A), further confirmed by the intensity profiles acquired in the 2D single-cell along an ideal arrow entirely sectioning the cell nucleus (inset in Fig. 3B): **c-exNDI** and 1H6 signals displayed partial overlapping profiles (red and green lines, respectively, Fig. 3B). The overlap coefficient^41^ was 0.77 out of 1.00 (Fig. 3B). The compound also showed signal peaks not colocalizing with 1H6. However, this behaviour is compatible with the intrinsically different nature of **c-exNDI** and 1H6. The former, being a small molecule of 540.6 Da, has a wider distribution than the latter, a protein of about 150 kDa, likely more hindered in its cellular distribution. Since we showed that G4 binding induced a red-shift in the emission maximum of **c-exNDI** *in vitro*, we checked whether this effect was detectable also in cells: thus emission signals at 601–609 nm and 609–617 nm were collected and compared. A fluorescence increase of 10.0 ± 0.6% at the emission interval 609–617 nm compared to that at 601–609 nm was observed (Fig. S12, ESI†). It is important to note that, in the absence of red shift, lower fluorescence intensity at 609–617 nm would be expected since the emission spectra of the free **c-exNDI** rapidly decreases at this wavelength interval by roughly 30%. The observed behaviour is consistent with the red shift and increase in emission upon **c-exNDI** binding to G4s obtained *in vitro*.

**Fig. 3:**
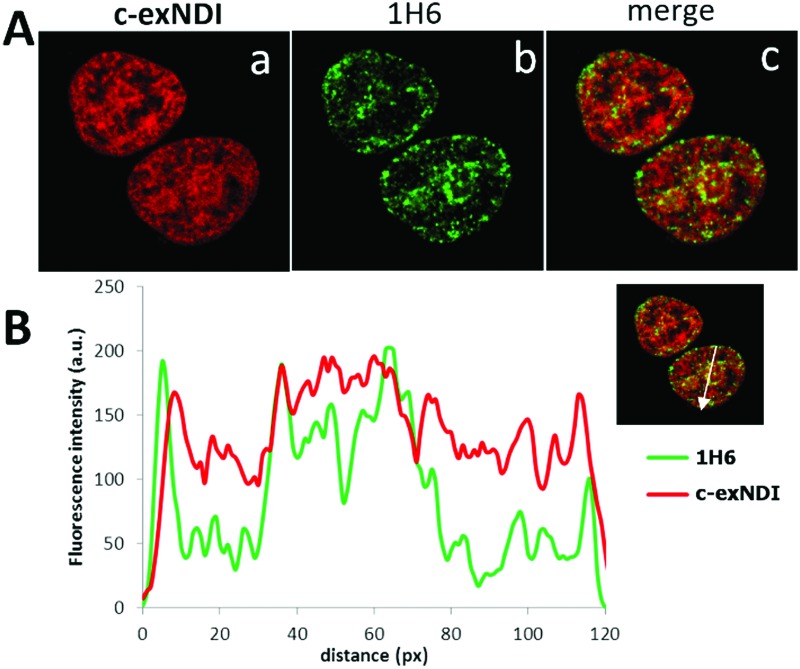
Colocalization of **c-exNDI** and G4s by confocal microscopy. (A) Cells were incubated with **c-exNDI** (red signal, panel a) and with the anti-G4 antibody 1H6 (green signal, panel b). The image on the right (merge) shows **c-exNDI** (red) and G4 (green) overlapping. (B) Intensity profiles of **c-exNDI** (red) and G4s (green) obtained using ImageJ software, along an ideal straight line (white) crossing the nucleus of a representative cell (right inset).

**c-exNDI** was mainly clustered in subnuclear organelles, whose appearance was compatible with that of nucleoli. To confirm the identity of these subnuclear compartments, fibrillarin, a component of nucleolar snRNPs, was used as a marker along with **c-exNDI**. Fibrillarin stained the same subnuclear bodies as **c-exNDI**, confirming the preferential nucleolar localization of the compound (Fig. 4A). Nucleolin (NCL) is a G4-binding protein, which is mainly localized in the nucleolus.^42^ It has been previously shown that treatment with quarfloxin (QFX), a potent G4 ligand, induced displacement of NCL, without affecting fibrillarin.^43^ We thus compared the ability of **c-exNDI** and QFX to displace NCL from the nucleoli. At 5 μM both **c-exNDI** and QFX induced a relocalization of NCL to the nucleoplasm outside the nucleoli (Fig. 4B, panels c and c′). In contrast, neither of them affected fibrillarin distribution (Fig. 4B, panels d–f and d′–f′).

**Fig. 4:**
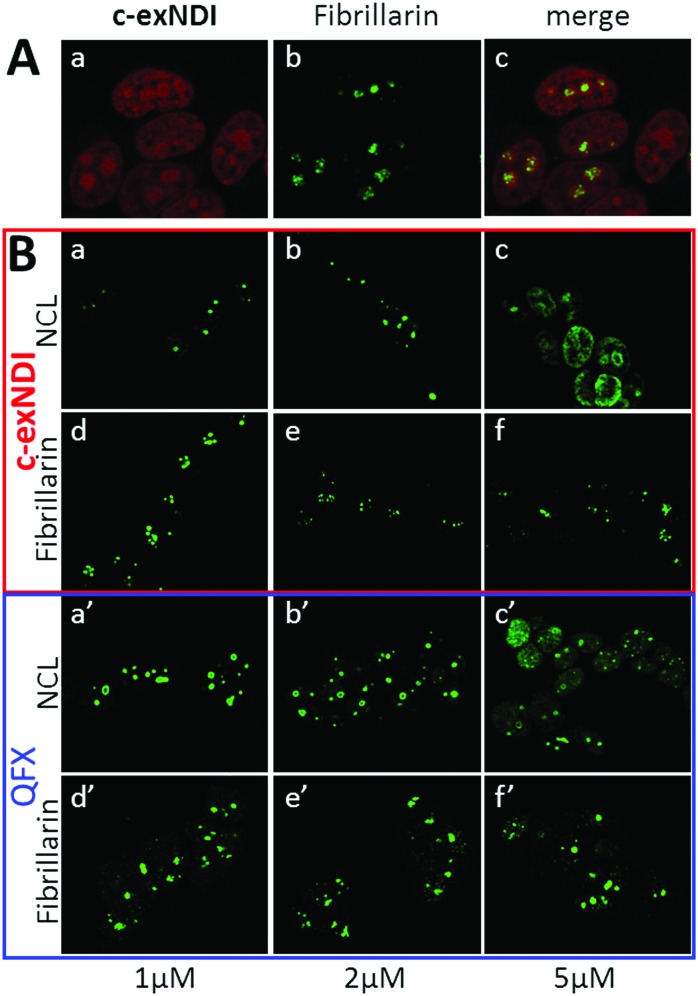
Cellular localization and targeting of **c-exNDI**. (A) Nucleolar localization of **c-exNDI**. Cells treated with **c-exNDI** (red signal, panel a) were incubated with an anti-fibrillarin antibody (green signal, panel b). Colocalization is shown in panel c. (B) **c-exNDI**-mediated displacement of the G4 binding protein nucleolin from the nucleoli. Cells were treated with increasing concentrations of **c-exNDI** (panels a–f) or quarfloxin (QFX) (panels a′–f′). Nucleolin (NCL) and fibrillarin behaviour upon treatment with the **c-exNDI** or QFX was visualized by staining the cells with anti-nucleolin (panels a–c and a′–c′) and anti-fibrillarin (panels d–f and d′–f′) antibodies.

In conclusion, we proved that aggregated non-emitting **c-exNDI** becomes selectively monomeric and fluorescent when bound to G4s. In particular, *in vitro* evaluation highlighted a maximal emission enhancement in the presence of hTel22 G4 in K^+^ solution and complexation constants confirmed a preferential binding to G4s with respect to dsDNA. In cells, **c-exNDI** rapidly and preferentially localized in the nuclei, showing co-localization with the 1H6 G4-specific antibody. Formation of the G4–**c-exNDI** complex in cells was also supported by the typical red-shifted emission observed *in vitro.* Not only specific localization at the nucleoli, but also binding to nucleolar G4s was confirmed by comparative nucleolin displacement assays. These collective results may be the first steps to achieve *in situ* detection of spots of selected DNA G4s in cell nuclei by a small fluorescent dye.

We thank Prof. P. M. Lansdorp for providing the 1H6 antibody and Prof L. H. Hurley for the gift of quarfloxin. This work was supported by the European Research Council (ERC Consolidator grant 615879) to S. N. R. and M. F., the Bill and Melinda Gates Foundation (GCE grants OPP1035881, OPP1097238) to S. N. R, and the Italian Association for Cancer Research [AIRC grant 14708] to M. F.
