# Supplementary material for: A red-NIR fluorescent dye detecting nuclear DNA G-quadruplexes: in vitro analysis and cell imaging
Source: Chem Commun (Camb). 2017 Jan 23;53(14):2268–71. doi: 10.1039/c6cc08492c (PMC5471928; doi:10.1039/c6cc08492c)
Supplement: Supplementary file 1 [file CC-053-C6CC08492C-s001.pdf]

Electronic Supplementary Material (ESI) for ChemComm.
This journal is © The Royal Society of Chemistry 2017

A red-NIR fluorescent dye detecting nuclear DNA G-quadruplexes: in vitro analysis and cell imaging

F. Doria,[a] M. Nadai,[b] M. Zuffo[a], R. Perrone[b], M. Freccero[a] and S. N. Richter*[b]*

[a] Department of Chemistry, University of Pavia, V.le Taramelli 10, 27100 Pavia, Italy.
[b] Department of Molecular Medicine, University of Padua, via Gabelli 63, 35121 Padua, Italy.

F. Doria and M. Nadai, contributed equally to this work.

Table of Contents.

**Experimental**	S1-S2
**Scheme S1**. Structure of the tetracationic fluorescent standard	S3
**Fig. S1.** Absorption and emission spectra of **c-exNDI** in various solvents	S3
**Fig. S2.** Absorption and emission spectra of **c-exNDI** with solvent composition from water to neat THF.	S4
**Fig. S3.** Absorption and Emission of **c-exNDI** as a function of Temperature	S4
**Fig. S4.** Absorption and Emission of **c-exNDI** as a function of pH	S5
**Fig. S5.** Absorption spectra of **c-exNDI** water solution at varying concentration and isodesmic fitting	S5
**Fig. S6.** Absorption and Excitation spectra of **c-exNDI**	S6
**Table S1.** Oligonucleotide sequences used in the present study	S6
**Table S2. c-exNDI** fluorescence quantum yields measured in the presence of NAs	S7
**Fig. S7.** Fitting of the fluorescence and absorption titration data	S7
**Table S3.** Stoichiometries and binding constants for the NA-**c-exNDI** complexes	S7
**Fig. S8.** Job plot analyses	S8
**Fig. S9.** Direct interaction of c-exNDI with hTel22 G4 measured by CD and Taq polymerase stop assay	S9
**Fig. S10.** Cell entry properties of the **c-exNDI**	S10
**Fig. S11**. Fluorescence microscopy images of **c-exNDI**-treated cells acquired in two different emission windows.	S11
**Fig. S12**. Binding of **c-exNDI** to intracellular DNA. Cells treated with RNase or DNase.	S11

Experimental

Materials and methods.

Reagents, solvents and chemicals were purchased from Alfa Aesar or Sigma-Aldrich and were used as supplied without further purification. **c-exNDI** was synthesised and purified according to published procedure.[S1] DNA and RNA oligonucleotides were purchased from Sigma Aldrich. pH was measured using a Thermo Orion 420+ pHmeter (Hamilton combined glass electrode). UV/Vis spectra were recorded on a JASCO V-550 UV/VIS spectrophotometer. Emission experiments were run on a Perkin Elmer LS-65 fluorometer. The temperature was controlled (±0.1°C) with Cary single cell peltier systems. Quartz cuvettes of 0.4 and 1 cm path length were used. DNAse free water was used throughout all the experiments involving oligonucleotides. The water stock solutions of oligonucleotides ($2x10^{-3}$-$5x10^{-4}$ M) and the probe ($5x10^{-3}$ M) and the DMF stock solutions of the probe ($5x10^{-3}$ M) were stored at -20°C. The appropriate dilutions were performed with water, 1M KCl or NaCl and 1M TRIS-HCl (pH 7.4) aqueous solutions. Annealing of G4 forming oligonucleotides was performed heating them in water ($1x10^{-1}$ M KCl or NaCl and $1x10^{-2}$ M TRIS-HCl, pH 7.4) at 95°C for 5 min and then letting them cool down to room temperature overnight. Heating was performed with a termoblock (Accublock by Labnet). If necessary, they were stored at 4°C.

Spectroscopic studies.

Deprotonated **c-exNDI** from DMF stock solution was diluted with the appropriate solvents to record preliminary spectra. $2x10^{-5}$ M solutions were prepared in neat water and THF and mixed in the appropriate proportions before recording absorption and emission spectra. For the temperature dependent absorption studies, solutions at the appropriate concentration were cooled down to 20°C and then heated up to 90°C. Spectra were recorded after each 5°C interval. For the spectrophotometric pH studies, **c-exNDI** was diluted at the appropriate concentration in $1x10^{-3}$ M buffers (either phosphoric acid - sodium dihydrogen phosphate or sodium dihydrogen phosphate - disodium monohydrogen phosphate). For spectrofluorimetric pH studies, **c-exNDI** was diluted the appropriate concentration in $1x10^{-3}$ M sodium borate buffer (initial pH 2) and the pH was increased adding aliquots of 0.1 M NaOH until pH 12 was reached.

Titrations and fluorescence quantum yield measurements.

5x10^{-6} M solutions of **c-exNDI** were titrated with 1x10^{-4} M solutions of NAs (Table 1). Absorption and emission (λ_{exc} = 605 nm) spectra were recorded after each NA addition and normalized molar absorptivity and fluorescence were plotted as a function of the NA concentration. Data were fitted according to 1:1 and 1:2 models[S2] (1 NA and 1 or 2 **c-exNDI**) with GraphPad 7.0 by Prism. Fluorescence quantum yields were measured using N,N′-bis[(trimethylamino)propylamino]-2,6-[(trimethylamino)propylamino]-1,4,5,8-naphthalene tetracarboxylic bisimide tetrachloride as a reference (Φ_{ref} = 17%)[S3] and exciting at 520 nm. The emission spectra were integrated between 540 and 800 nm. Fluorescence quantum yields were measured according to the following formula, correcting the values for sample and reference absorption at the excitation wavelength:

$$\Phi_f = \Phi_{ref} * \frac{Area_{sample}}{Area_{ref}} * \frac{A_{ref}}{A_{sample}}$$

Confocal microscopy

Human embryonic kidney (HEK) 293T cells (ATCC # CRL-3216) were grown in DMEM (Gibco, Thermo Fisher Scientific, Waltham, MA, USA) supplemented with 10% heat-inactivated fetal bovine serum (FBS, Gibco). Cells were maintained as a monolayer in the logarithmic growth phase at 37 °C in a 5% CO_2 humidified atmosphere. Cells were seeded onto glass coverslips in 6 well plates and incubated overnight; the next day they were treated with **c-exNDI** 1μM for 30 min at 37 °C, fixed with 2% paraformaldehyde in phosphate-buffered saline (PBS) for 20 min and then permeabilized with 0.5% Tween 20 in PBS. Cells were treated with 40 μg/ml RNaseA (Invitrogen) or 200 units DNase I (Invitrogen) and then blocked with BlockAid (Invitrogen).

For the internalization assay, cells were treated with **c-exNDI** (1μM) for 2.5, 5, 7.5, 10, 15, 30 min and then fixed as described above.

For G4 visualization, cells were incubated with monoclonal antibody 1H6 (kindly provided by P. Lansdorp, European Research Institute for the Biology of Ageing, University of Groeningen, the Netherlands), specific for G4 DNA, for 2 h at r. t.[S4] For nucleolin and fibrillarin visualization, cells were incubated with C23 antibody (H-250) (SantaCruz Biotechnology) or with anti-fibrillarin antibody (38F3) (Abcam) for 1 h at 37 °C. After washing in PBS-Tween, cells were incubated with Alexa 488 anti-mouse IgG or Alexa 488 anti-rabbit IgG (Invitrogen), and mounted with Glycergel Mounting Medium (Dako).

Cells were treated with 1, 2 and 5 μM Quarfloxin (CX-3543) (kindly provided by Laurence H. Hurley, Tetragene and University of Arizona) for 2 h.[S5]

Images were acquired with Leica TCS SP2 confocal microscope: for **c-exNDI,** images were visualized at 543 nm excitation wavelength and 609-617 nm emission range; for G4, nucleolin and fibrillarin at 488 nm excitation wavelength and 500-530 nm emission range were applied.

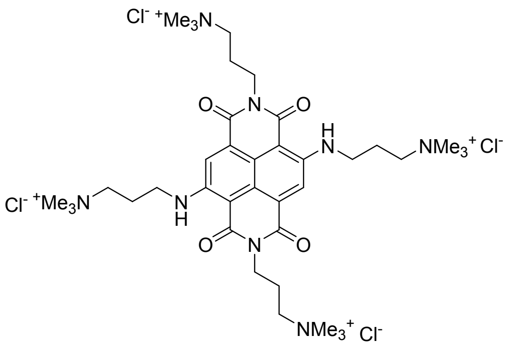

Scheme S1. Structure of N,N′-bis[(trimethylamino)propylamino]-2,6-[(trimethylamino) propylamino]-1,4,5,8-naphthalenetetracarboxylic bisimide tetrachloride, which has been used as fluorescent standard (Φ=0.17).

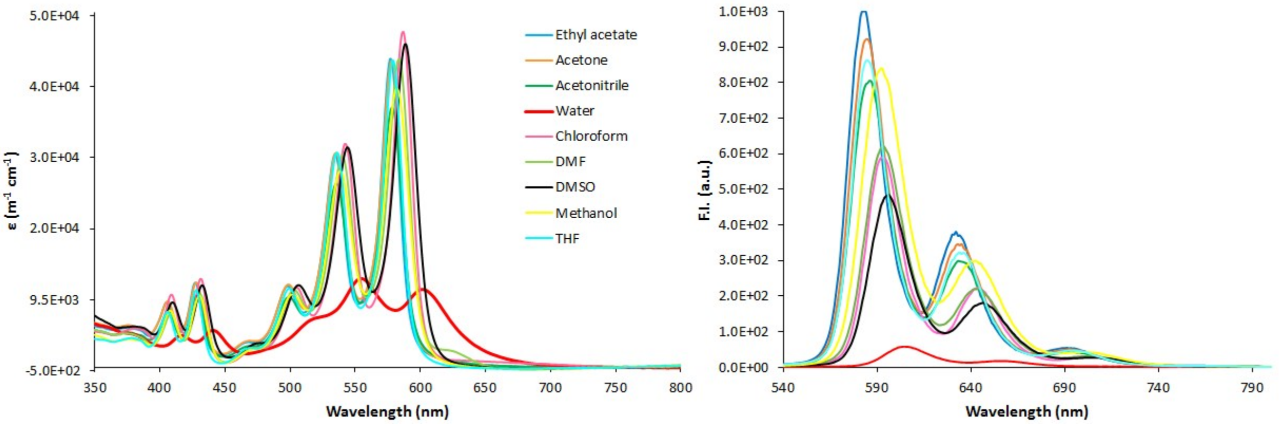

Fig. S1. Absorption (2x10^{-5} M) and emission (5x10^{-6} M, λ_{exc} = 520 nm) spectra of **c-exNDI** solutions in water and various organic solvents (acetone, ethyl acetate, acetonitrile, chloroform, dimethylformamide, dimethylsulfoxide, methanol, tetrahydrofuran).

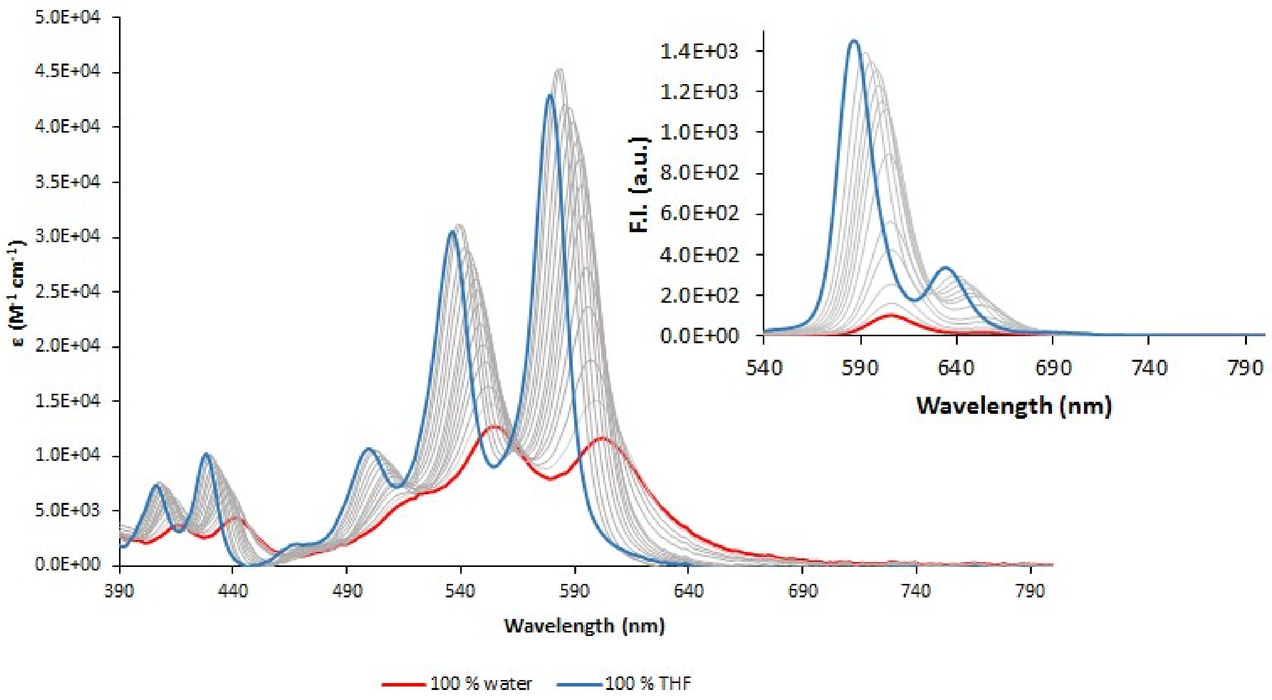

Fig. S2. UV-Vis (2x10^{-5} M) and emission (5x10^{-6} M, λ_{exc} = 520 nm, inset) spectra of **c-exNDI** solutions in mixtures of H_2O/THF at 25°C showing disaggregation of the **c-exNDI** aggregate in water (red line) to the monomer in neat THF (blue line).

.

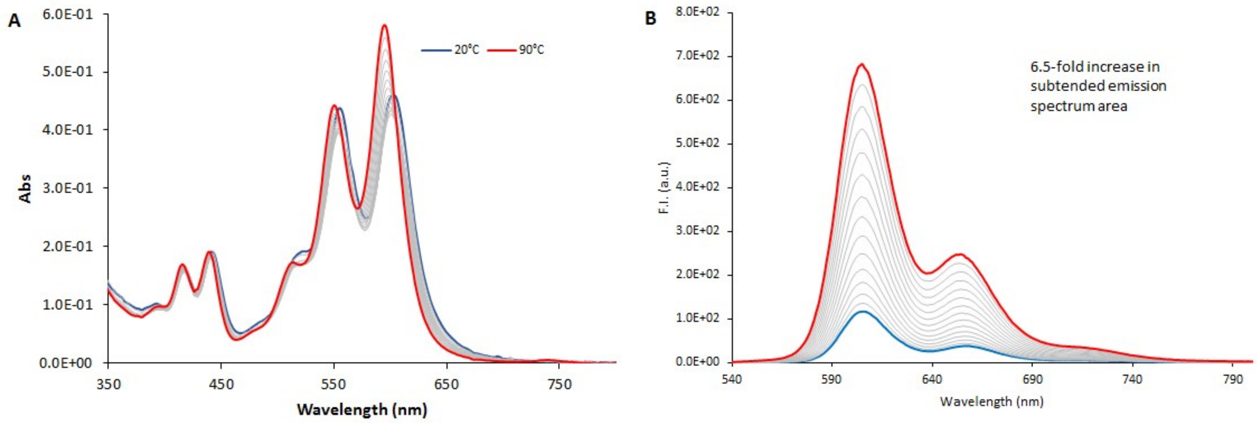

Fig. S3. A) Absorption of a 2x10^{-5} M solution of **c-exNDI** in water recorded while varying the temperature between 20 and 90°C; B) emission of a 5x10^{-6} M (λ_{exc} = 520 nm) solution of **c-exNDI** in water recorded in the same conditions.

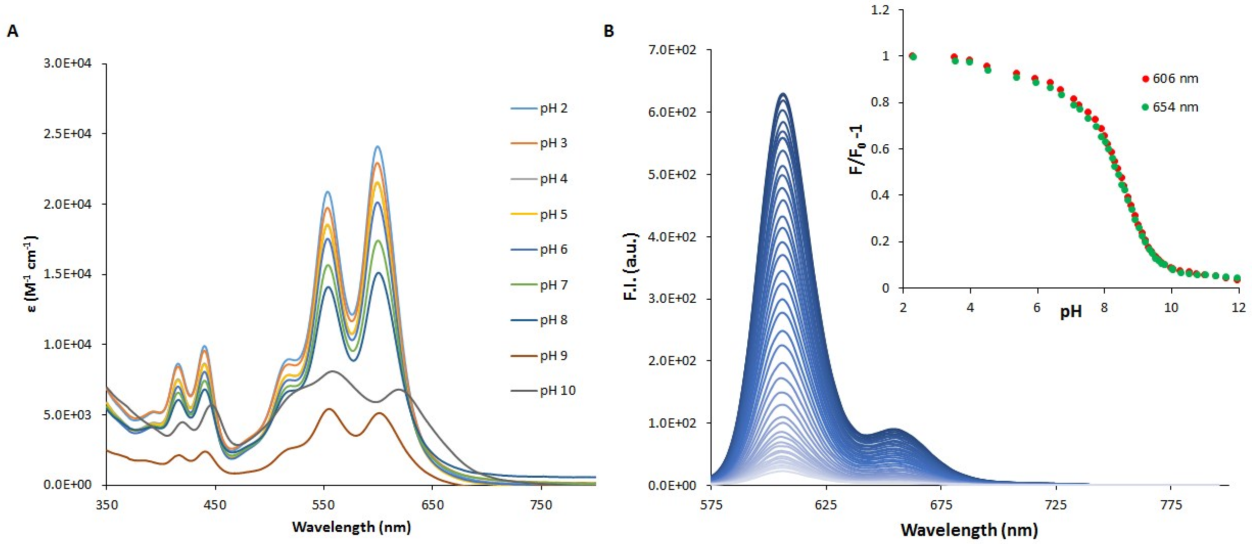

Fig. S4. A) Absorption spectra of 2.5x10^{-5} solutions of **c-exNDI** in water at different pH values, between 2 and 10. B) Emission spectra of a 5x10^{-6} solution of **c-exNDI** in water (λ_{exc} = 540 nm) obtained varying the pH from 2 to 12. Inset: normalized fluorescence intensity in the maxima plotted as a function of pH to calculate the pKa value (8.38±0.03, obtained with Sigmaplot). The pKa values of the two tertiary amine groups are not distinguishable and cannot be existimated potentiometrically (the compound precipitates above pH 8 in the concentration range necessary for such analyses).

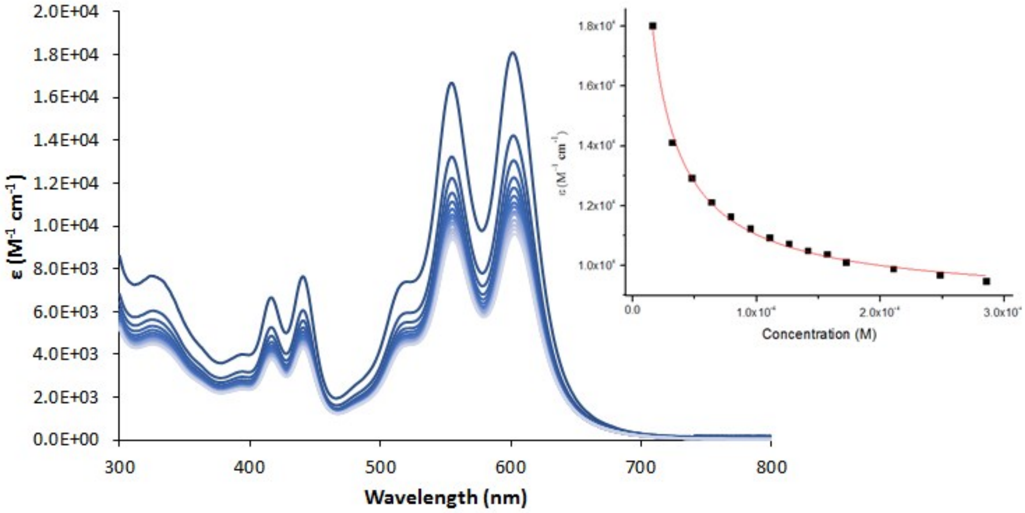

Fig. S5. Absorption spectra of **c-exNDI** water solution (1x10^{-1} M KCl, 1x10^{-2}M TRIS-HCl, pH 7.4) measured at increasing concentration (1.5x10^{-5}-3x10^{-4} M) of the compound. Molar absorbivity coefficient at 595 nm plotted as a function of probe **c-exNDI** concentration. Data were fitted according to an isodesmic model (OriginLab).

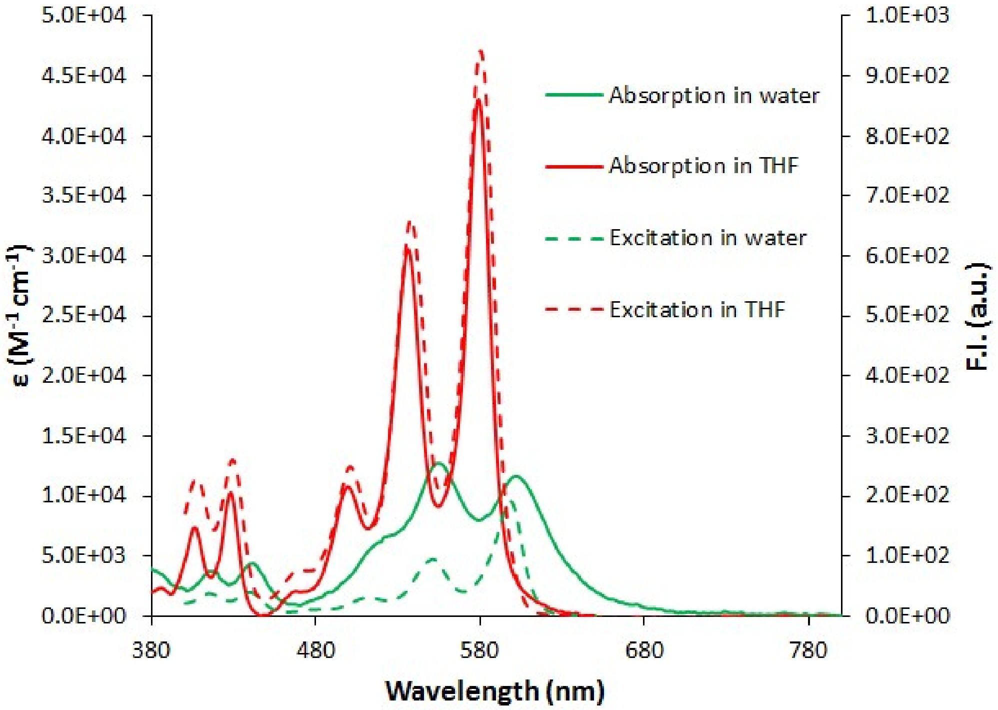

Fig. S6. Absorption and excitation spectra of **c-exNDI** in water and THF solution, measured between 400 and 800 nm (λ_{em}= 600 nm).

Tab. S1. Oligonucleotide sequences used in the present study

NA	Sequence	Topology
hTel22 (K^+)	5′-d(AGGGTTAGGGTTAGGGTTAGGG)-3′	Hybrid
hTel22 (Na^+)	5′-d(AGGGTTAGGGTTAGGGTTAGGG)-3′	Anti-parallel
HRAS	5′-d(TCGGGTTGCGGGCGCAGGGCACGGGCG)-3′	Anti-parallel
TBA	5′-d(GGTTGGTGTGGTTGG)-3′	Anti-parallel
c-kit1	5′-d(AGGGAGGGCGCTGGGAGGAGGG)-3′	Parallel
c-kit2	5′-d(CCCGGGCGGGCGCGAGGGAGGGGAGG)-3′	Parallel
c-myc	5′-d(TGAGGGTGGGTAGGGTGGGTAA)-3′	Parallel
ssDNA	5′-d(GGATGTGAGTGTGAGTGTGAGG)-3’	Single strand
dsDNA	5′-d(CAATCGGATCGAATTCGATCCGATTG)-3’	Duplex

Tab. S2. Fluorescence quantum yields measured for **c-exNDI** ($5x10^{-6}$ M) in water ($1x10^{-1}$ M KCl or NaCl, $1x10^{-2}$ M TRIS-HCl buffer, pH 7.4) in the presence of $5x10^{-6}$ M of the NAs analysed in this study using as a reference the tetracationic NDI standard (**Scheme S1**).

NA	$\Phi_{fluorescence}$
**c-exNDI** - hTel22 (KCl)	0.24
**c-exNDI** - hTel22 (NaCl)	0.20
**c-exNDI** - HRAS	0.19
**c-exNDI** - TBA	0.17
**c-exNDI** - ssDNA	0.16
**c-exNDI** – c-myc	0.14
**c-exNDI** – c-kit1	0.14
**c-exNDI** – c-kit2	0.13
**c-exNDI** - dsDNA	0.09
**c-exNDI**	0.08

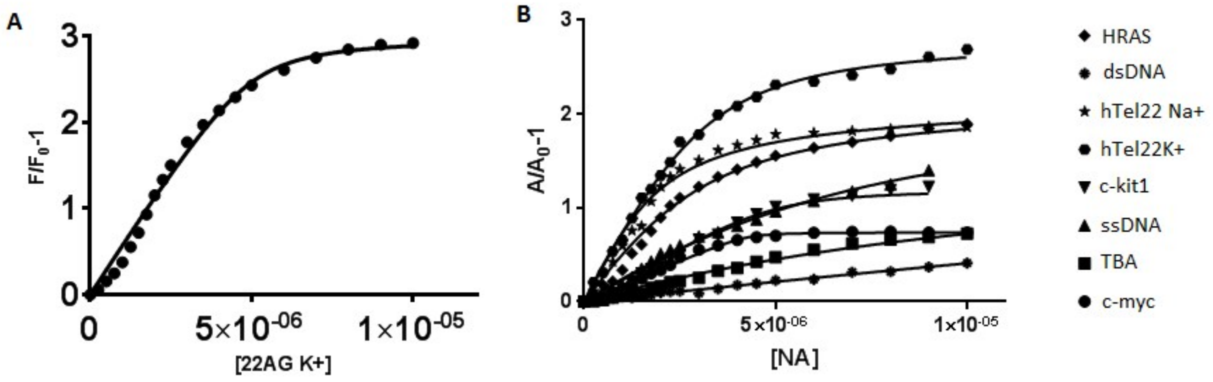

Fig. S7. A) Fitting of the fluorescence titration data (normalized fluorescence at 613 nm) of a $5x10^{-6}$ solution of **c-exNDI** (water, $1x10^{-1}$ M KCl, $1x10^{-2}$ M TRIS-HCl buffer, pH 7.4) with hTel22 G4 in K^+. Fitting was performed according to 1:1 model. B**)** Fitting of the fluorescence titration data (normalized absorption at 605-620 nm) of $5x10^{-6}$ solution of **c-exNDI** (water, $1x10^{-1}$ M KCl, $1x10^{-2}$ M TRIS-HCl buffer, pH 7.4) with hTel22 (in either K^+ or Na^+ solutions), HRAS, c-kit1, c-myc, TBA, dsDNA and ssDNA NAs. Fitting was performed according to 1:1 or 1:2 models, depending on Job plot indications and best fitting.

Tab. S3. Stoichiometries and binding constants for the NA-**c-exNDI** complexes. Binding constants were calculated from the fitting of *a*) emission data; *b*) absorption data

NA	Stoichiometry (NA:c-exNDI)	$K_{1:1}$ (M^{-1}) or $K_{1:2}$ (M^{-2})
hTel22 (K^+)	1:1	$5.34\pm0.03x10^{6}$ [a]
	1:1	$5.37\pm0.06x10^{6}$ [b]
hTel22 (Na^+)	1:2	$3.0\pm0.3x10^{11}$ [b]
HRAS	1:2	$3.0\pm0.3x10^{10}$ [b]
c-kit1	1:1	$4.82\pm0.04x10^{6}$ [b]
c-myc	1:1	$3.43\pm0.03x10^{7}$ [b]
TBA	1:1	$1.2\pm0.5x10^{5}$ [b]
ssDNA	1:1	$2.85\pm0.01x10^{5}$ [b]
dsDNA	1:1	$7.41\pm0.03x10^{3}$ [b]

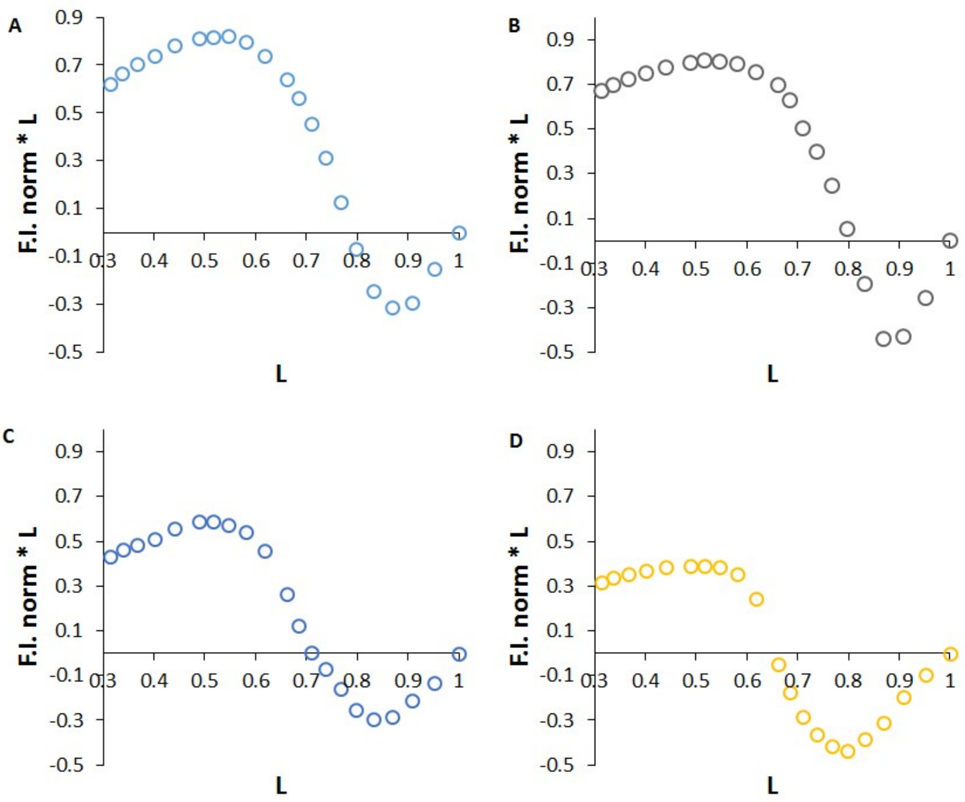

Fig. S8. Job plot analyses of fluorescence titration data of **c-exNDI** with A) HRAS; B) hTel22 (Na^+); C) c-kit1; D) c-myc. L = [**c-exNDI**]/([**c-exNDI**]+[G4]) and F.I. norm. = F/F_0-1 in the emission maximum.

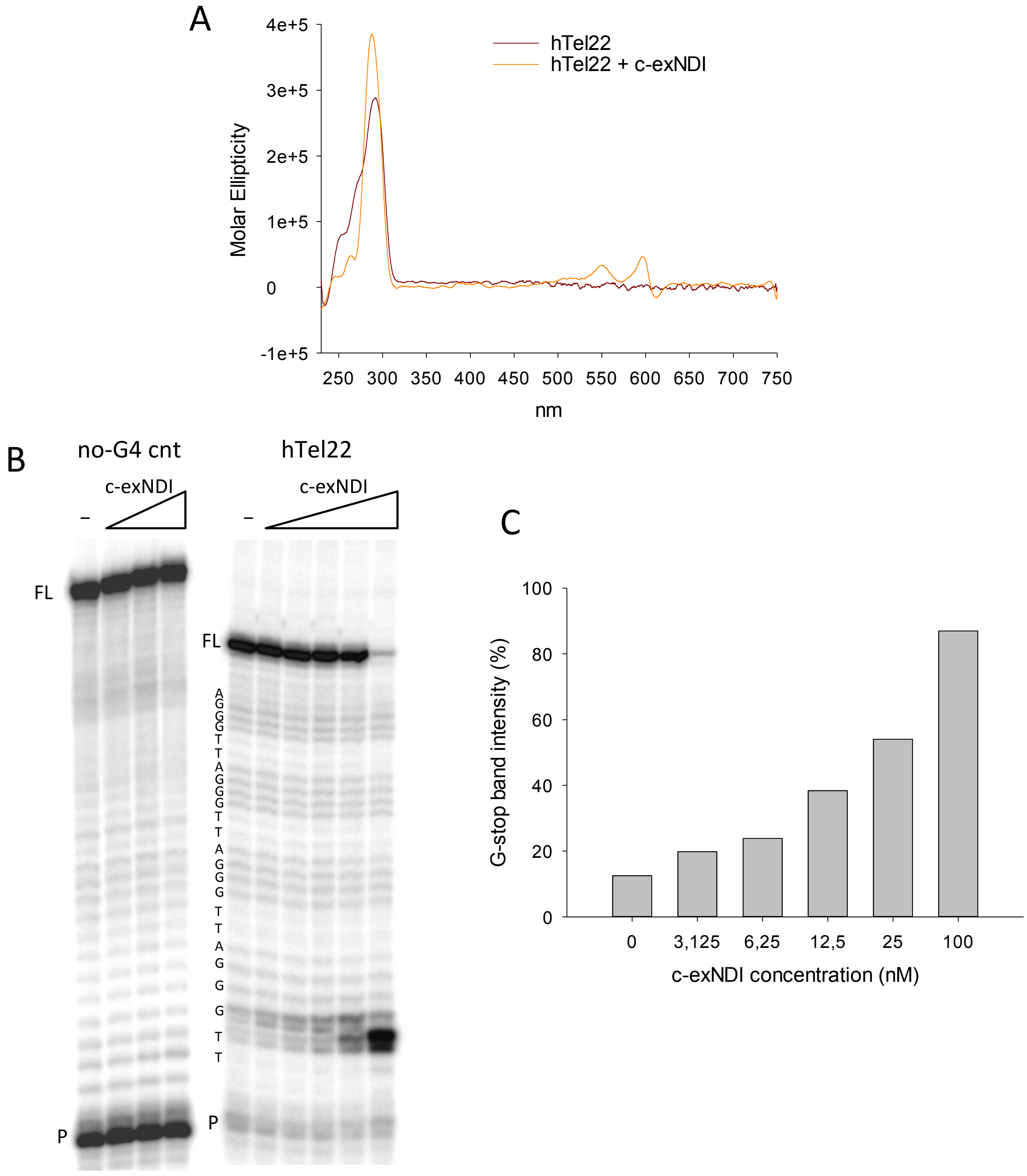

Figure S9: Direct interaction of **c-exNDI** with hTel22 G4 measured by CD and Taq polymerase stop assay. A) CD spectra of the G4-folded hTel22 oligonucleotide (4 µM) in the presence of KCl (100 mM) and with c-exNDI (16 µM). The c-exNDI binds the G4 hTel22 inducing a partial change in the G4 conformation and generating ICD bands in the UV/Vis absorption regions of the ligand. B) Taq polymerase stop assay in sequencing polyacrylamide gel of the G4-folded hTel22 template (hTel22) and a control sequence unable to fold into G4 (no-G4 cnt). In the hTel22 template a clear pausing site of the Taq polymerase is visible right before the most 3'-end G-tract of the oligonucleotide. P stands for primer and FL for full-length amplification product. The relevant sequence of hTel22 is shown on the left of the sequencing bands. C) Quantification of the stop bands in the hTel22 template observed in panel B. The band in the untreated sample (c-exNDI = 0) is arbitrarily set to 100%.

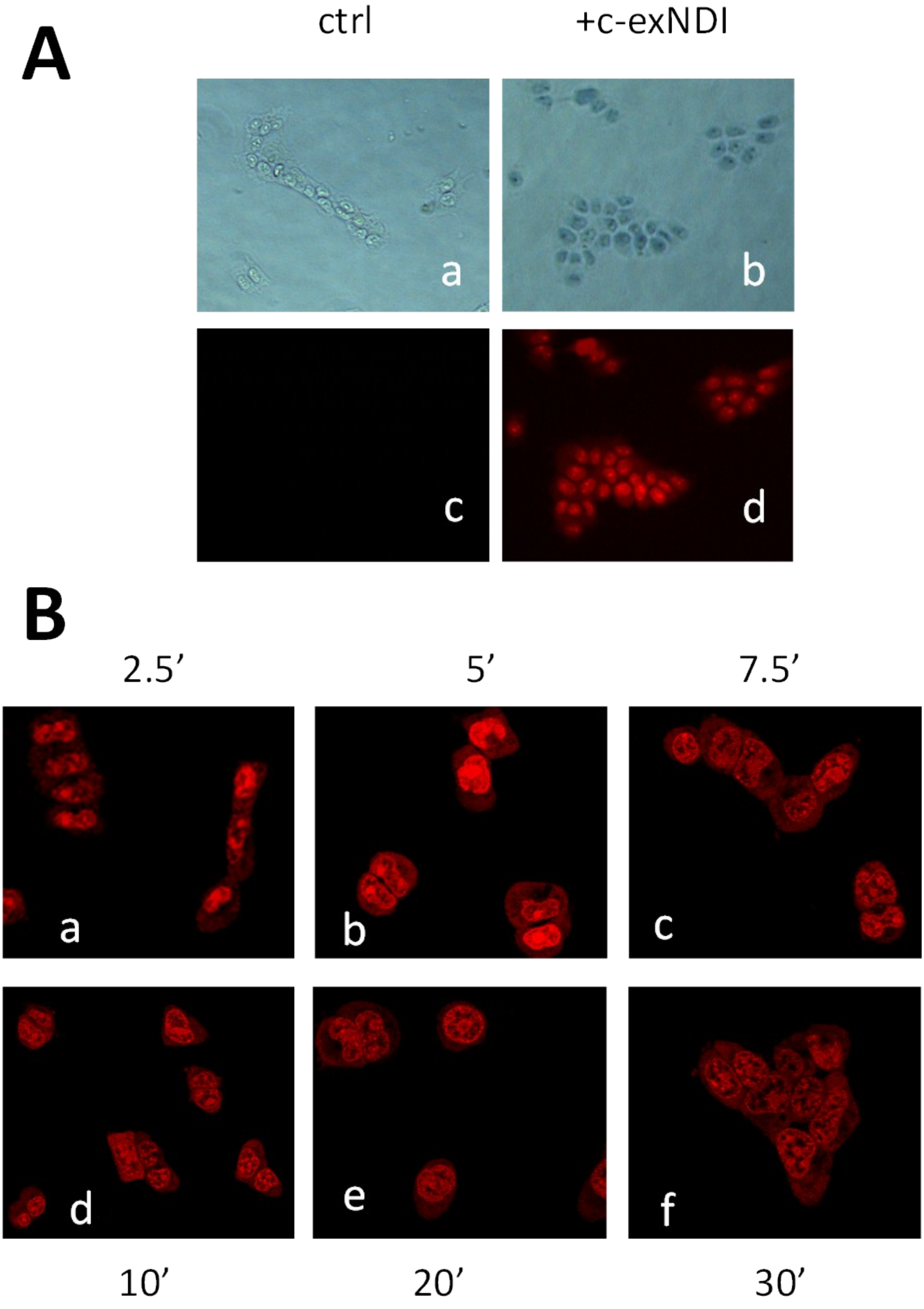

Fig. S10. Cell entry properties of the **c-exNDI**. A) Transmitted light (panels a and b) and fluorescence (panels c and d) microscopy analyses of **c-exNDI**-treated 293T cells. Panels a and c are control images of non-treated cells. B) Time-course of **c-exNDI** entry into the cells, monitored at 2.5 (a), 5 (b), 7.5 (c), 10 (d), 20 (e), and 30 (f) minutes of incubation by fluorescence microscopy.

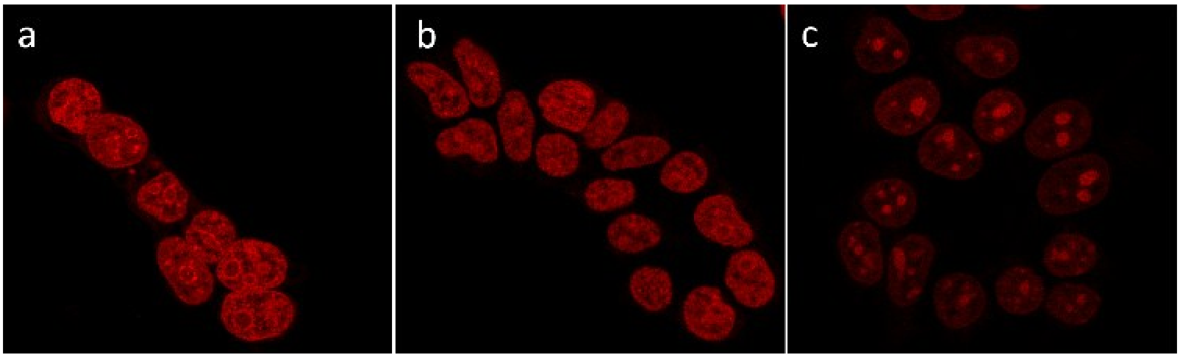

Fig. S11. Binding of **c-exNDI** to intracellular DNA. Cells were incubated with the **c-exNDI** and fixed (panel a), and treated with RNase (panel b) or DNase (panel c).

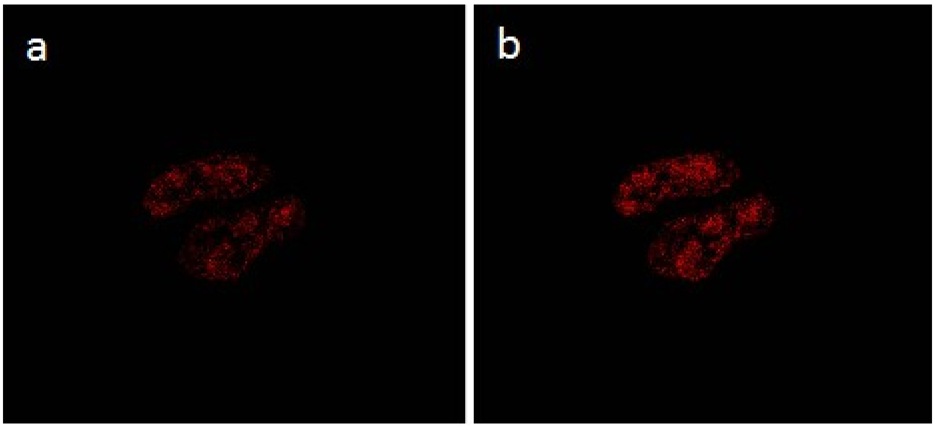

Fig. S12. Fluorescence microscopy images of 293T cells treated with **c-exNDI** and acquired upon excitation at 543 nm and emission at 601-609 nm (panel a) and 609-617 nm (panel b) intervals. Quantification of the signal intensity was performed with ImageJ software on a least 6 different cell acquisitions.

Supplementary references

S1. R. Perrone, F. Doria, E. Butovskaya, I. Frasson, S. Botti, M. Scalabrin, S. Lago, V. Grande, M. Nadai, M. Freccero and S. N. Richter, *J. Med. Chem.* 2015, **58**, 9639-9652.

S2. M. Zuffo, F. Doria, V. Spalluto, S. Ladame and M. Freccero, *Chem. Eur. J.*, 2015, **21**, 17596-17600

S3. F. Doria, I. Manet, V. Grande, S. Monti, and M. Freccero, *J. Org. Chem.* 2013, **78**, 8065-8073.

S4. A. Henderson, Y. Wu, Y. C. Huang, E. A. Chavez, J. Platt, F. B. Johnson, R. M. Brosh, Jr., D. Sen and P. M. Lansdorp, *Nucleic Acids Res.*, 2014, **42**, 860-869.

S5. D. Drygin, A. Siddiqui-Jain, S. O'Brien, M. Schwaebe, A. Lin, J. Bliesath, C. B. Ho, C. Proffitt, K. Trent, J. P. Whitten, J. K. Lim, D. Von Hoff, K. Anderes and W. G. Rice, *Cancer Res.*, 2009, **69**, 7653-7661
